# New Insights Into Chromomere Organization Provided by Lampbrush Chromosome Microdissection and High-Throughput Sequencing

**DOI:** 10.3389/fgene.2020.00057

**Published:** 2020-02-17

**Authors:** Anna Zlotina, Antonina Maslova, Olga Pavlova, Nadezda Kosyakova, Ahmed Al-Rikabi, Thomas Liehr, Alla Krasikova

**Affiliations:** ^1^ Saint Petersburg State University, Saint Petersburg, Russia; ^2^ Institute of Human Genetics, Jena University Hospital, Jena, Germany

**Keywords:** chromatin domains, lampbrush chromosome, chromomere, chromosome microdissection, chicken

## Abstract

Giant lampbrush chromosomes (LBCs) typical for growing oocytes of various animal species are characterized by a specific chromomere-loop appearance and massive transcription. Chromomeres represent universal units of chromatin packaging at LBC stage. While quite good progress has been made in investigation of LBCs structure and function, chromomere organization still remains poorly understood. To extend our knowledge on chromomere organization, we applied microdissection to chicken LBCs. In particular, 31 and 5 individual chromomeres were dissected one by one along the macrochromosome 4 and one microchromosome, respectively. The data on genomic context of individual chromomeres was obtained by high-throughput sequencing of the corresponding chromomere DNA. Alignment of adjacent chromomeres to chicken genome assembly provided information on chromomeres size and genomic boarders, indicating that prominent marker chromomeres are about 4–5 Mb in size, while common chromomeres of 1.5–3.5 Mb. Analysis of genomic features showed that the majority of chromomere-loop complexes combine gene-dense and gene-poor regions, while massive loopless DAPI-positive chromomeres lack genes and are remarkably enriched with different repetitive elements. Finally, dissected LBC chromomeres were compared with chromatin domains (topologically associated domains [TADs] and A/B-compartments), earlier identified by Hi-C technique in interphase nucleus of chicken embryonic fibroblasts. Generally, the results obtained suggest that chromomeres of LBCs do not correspond unambiguously to any type of well-established spatial domains of interphase nucleus in chicken somatic cells.

## Introduction

In highly extended chromosomes, such as polytene chromosomes, lampbrush chromosomes (LBCs), and pachytene chromosomes, a chromomere is defined as a universal unit of chromatin packaging (Vlad and Macgregor, 1975). While our understanding of structure and function of chromomeres in polytene chromosomes has considerably grown in recent years, chromomere organization in LBCs still remains poorly understood.

Chromomeres of lampbrush chromosomes, being typical for animal growing oocytes, are regarded as condensed and apparently transcriptionally inactive chromatin domains (Vlad and Macgregor, 1975; Macgregor, 2012). LBC chromomeres can be seen in both fixed and living chromosome preparations. An array of chromomeres constitutes an axis of each LBC, with neighboring chromomeres being connected by thin decondensed chromatin threads (interchromomeric fibers). Generally, chromomeres are unevenly distributed along the chromosome axis: arrays of massive and prominent chromomeres alternate with regions of small and medium-sized ones (Callan, 1986; Rodionov et al., 2002; Galkina et al., 2006). Besides, apart from chromomeres with numerous pairs of extended loops, there are some chromomeres lacking recognizable loops. Since chromomeres constantly appear in the same positions one can develop cytological maps reflecting the number, size, and general pattern of distribution of chromomeres along LBC's axes (Galkina et al., 2005; Galkina et al., 2006; Daks et al., 2010; Zlotina et al., 2012). One of the notable examples is LBC W of the domestic chicken that consists of seven distinct chromomeres (Solovei et al., 1993). From structural point of view LBC chromomeres are thought to represent a rosette of microloops, which are connected by protein clips at their bases. In particular, cohesin and condensin complexes that were found in LBC chromomeres can serve as such longitudinal and transverse clips (Beenders et al., 2003; Krasikova et al., 2005; Austin et al., 2009). Intriguingly, LBC axes lack any linker histones H1 (Hock et al., 1993).

Being compact chromatin domains, the overwhelming majority of chromomeres are enriched with epigenetic landmarks typical for inactive chromatin: 5-methylcytosine-modified DNA and the methylated DNA-binding protein MeCP2, histone H3 trimethylated at lysine 9 or lysine 27, as well as heterochromatin protein HP1β (Krasikova et al., 2009; Morgan et al., 2012; Krasikova and Kulikova, 2017). Certain chromomeres are less compact and looser in appearance and also comprise some amount of hyperacetylated histone H4 (Sommerville et al., 1993), which can be explained by transcriptional activity of certain microloops being a part of a chromomere. Nevertheless, while there is some data on overall structure, protein composition, and epigenetic status of LBC chromomeres, their genomic context has not been a focus of previous studies.

Morphologically discrete chromomeres can be mechanically dissected from a single copy of LBC by glass needles. Moreover, DNA fragments from individual isolated chromomeres can be deciphered by one of the next generation sequencing (NGS) approaches and assigned to certain regions in the reference genome assembly (Zlotina et al., 2016).

To extend our knowledge on chromomere organization and genomic context, we performed microdissection of all prominent chromomeres from lampbrush macrochromosome 4 and one of the microchromosomes in a chicken lampbrush chromosome set. The data on cytogenetic and genomic features of individual chromomeres were obtained by high-resolution fluorescence *in situ* hybridization (FISH) and high-throughput sequencing procedure. Finally, LBC chromomeres were compared with chromatin domains earlier identified by Hi-C technique in interphase nucleus of chicken embryonic fibroblasts. Generally, the results obtained in the present study suggest that chromomeres of LBCs do not correspond unambiguously to any type of well-established chromatin domains of interphase nucleus of somatic cells.

## Materials and Methods

### Chromosome Preparation and Needle-Based Microdissection Procedure

Chicken lampbrush chromosomes (LBCs) were manually isolated from growing oocytes, fixed in 2% formaldehyde and dehydrated as described elsewhere (https://projects.exeter.ac.uk/lampbrush/). Mitotic metaphase chromosomes were obtained from chicken embryonic fibroblasts according to standard protocols. All institutional and national guidelines for the care and use of laboratory and farm animals were followed. The animal studies received approval #131–03-2 of the Ethical committee of Saint-Petersburg State University.

Glass needle-microdissection of LBC chromomeres was performed according to the previously published protocol with some modifications (Zlotina et al., 2016; Zlotina et al., 2019). In brief, individual chromomeres were dissected one after another along the length of macrochromosome 4 and one of the microchromosomes under phase contrast microscopy. The microdissected fragments were transferred into micropipettes with a collection drop solution (30% glycerol, 10 mM Tris/HCl, pH 7.5, 10 mM NaCl, 0.1% SDS, 1 mM EDTA, 0.1% Triton X-100, 1.44 mg/ml proteinase K) followed by incubation at 60°C for 1–2 h. Primary amplification of the isolated DNA material was performed using DOP-PCR (degenerate oligonucleotide-primed PCR) with a degenerate universal primer 5′-CCG ACT CGA GNN NNN NAT GTG G-3′ as previously described (Zlotina et al., 2016).

### Fluorescence *In Situ* Hybridization

#### Probes Preparation

The primary DOP-PCR products were differentially labeled with biotin or digoxigenin by PCR with the same degenerate primer (for details see Zlotina et al., 2016). Labeled PCR products were dissolved in a standard hybridization buffer (50% deionized formamide [ICN], 2×SSC, 10% dextran sulphate [Sigma]) to a final concentration of 20–40 ng/μl with a 50-fold excess of salmon sperm DNA (Invitrogen).

#### *In Situ* Hybridization

The obtained FISH-probes were applied to mitotic metaphase and LBCs. Metaphase chromosomes were pre-treated with 0.01% pepsin and post-fixed with 1% formaldehyde in 1×PBS. FISH on LBCs was performed according to a DNA/(DNA+RNA) hybridization protocol without any pretreatments. Chromosomes and DNA-probes were co-denatured on a slide under a coverslip at 78°C for 5 min followed by hybridization at 37°C in a humid chamber for 16–20 h. Post-hybridization washings included two changes of 0.2×SSC at 60°C and two changes of 2×SSC at 45°C. Avidin-Alexa 488 (Molecular Probes Inc.) and mouse antibody against digoxigenin conjugated with Cy3 (Jackson ImmunoResearch Laboratories) were used to detect biotin- and digoxigenin-labeled probes, respectively. To amplify the signals, we performed an additional incubation with biotinylated anti-avidin (Vectorlabs) followed by the second round of incubation with avidin-Alexa 488 for biotin-labeled probes, and incubation with Cy3-conjugated goat anti-mouse IgG+IgM (H+L) (Jackson ImmunoResearch Laboratories) for digoxigenin-labeled probes. All preparations were dehydrated, air-dried, and mounted in antifade solution containing 1 μg/ml 4,6-diamidino-2-phenylindole (DAPI).

### DNA-Library Preparation and High-Throughput Sequencing

The DNA-library preparation was performed according to the manufacturer's recommendations with some modifications (Ion Torrent, Life Technologies). In particular, primary DOP-PCR products of the dissected material were re-amplified and barcoded using a panel of Ion-Torrent primers. Quality and quantity of the fragments were evaluated by high-resolution capillary electrophoresis using Shimadzu MultiNA (Japan). In average, fragment length distribution was 150–350 bp with a target pick at ~200 bp. To get rid of dimers of primers, the samples were purified using magnetic beads Agencourt AMPure XP (Beckman Coulter) followed by a capillary electrophoresis analysis. Final concentrations of the DNA-libraries were assessed using Qubit 2.0 Fluorometer (Invitrogen/Life Technologies USA), after that all samples were diluted to ~ 26 pM and equimolarly pooled. Sequencing run was carried out with Ion Torrent PGM genome analyzer (Life Technologies); single-end sequencing was performed. Procedures of emulsion PCR, Ion Sphere Particle Enrichment, and loading of Ion 318 Chip v2 were carried out according to the manufacturer's instructions.

### Sequencing Data Processing and Analysis

The sequencing data was processed and analyzed using the web-based bioinformatic platform Galaxy (https://usegalaxy.org/, Giardine et al., 2005) as earlier described (Zlotina et al., 2016). In brief, input files were converted to an appropriate FASTQ format using sff converter (version 1.0.1) and FASTQ Groomer (version 1.0.4) tools, after that the quality of the data was evaluated using the FastQC tool. To get rid of terminal adapter sequences and remove poor quality base calls from the end, the sequence reads were trimmed and filtered by length. The reads were mapped to chicken reference genome assembly (https://www.ncbi.nlm.nih.gov/genome/111, Gallus_gallus-5.0) using a short-read aligner Bowtie2 (Langmead and Salzberg, 2012). The data visualization and analysis were carried out with genome browser Integrative Genomics Viewer (IGV) (Robinson et al., 2011). The mapped chromosome regions were also evaluated with regard to some genome characteristics (such as gene-density, repeats content) using corresponding imported tracks downloaded from the UCSC Genome Browser (http://genome.ucsc.edu).

Coordinates of topologically associated domains (TADs) and A/B compartments in chicken embryonic fibroblasts (CEF) were imported from http://icg.nsc.ru/ontogen/ (Fishman et al., 2019). For comparative analysis the chromomere borders were defined according to the alignment of sequenced reads to the reference genome and by excluding single reads distant from the main cluster of reads. The chromomeres with ambiguous borders were excluded from the analysis. The comparison of genomic coordinates of chromomeres and A/B compartments was performed by visual matching and by counting switches of the compartment type within 500 kb distance from left and right chromomere borders. The number of TADs per chromomere was counted using JuiceBox heatmaps and the defined chromomere borders.

## Results

### Microdissection of Individual Chromomeres From Chicken Lampbrush Chromosomes and Their Mapping on Mitotic Metaphase Chromosomes

To analyze the genomic organization of LBC chromomeres, we applied mechanical microdissection to chicken lampbrush chromosomes followed by preparation of DNA-libraries of isolated chromomeres. In particular, all prominent chromomeres were dissected one by one along the chicken lampbrush macrochromosome 4 starting from the q-ter chromosome region: chromomeres ##1–31 (**Supplementary Figure S1**). In chicken karyotype, chromosome 4 has an interesting evolutional background being a result of centric fusion of ancestral macrochromosome 4 (GGA4q) and a microchromosome (GGA4p) (Shibusawa et al., 2004; Griffin et al., 2007). Additionally, we isolated chromomeres constituting one of chicken microchromosomes in the lampbrush form.

**Figure 1:**
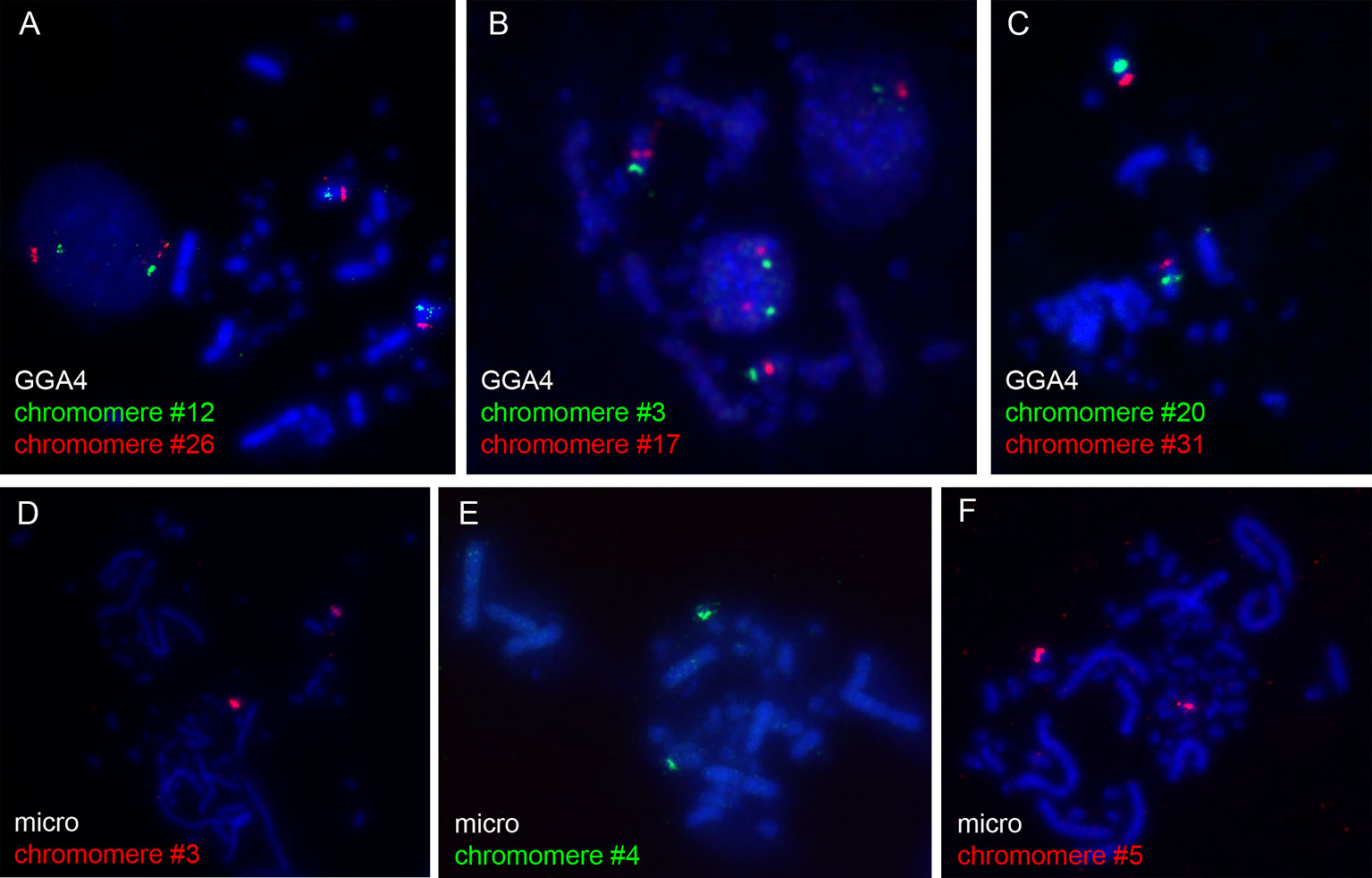
FISH-mapping of microdissected lampbrush chromosome chromomeres on chicken metaphase chromosomes. Examples of FISH with DNA material of individual chromomeres on metaphase macrochromosome 4 **(A–C)** and microchromosome 11 **(D–F)**. Chromomere ID numbers are indicated. Chromosomes are counterstained with DAPI.

The microdissected material was used for preparation of DNA probes for FISH. To verify the quality and specificity of the dissected samples, the DNA fragments were mapped on chicken metaphase chromosomes. Among 31 DNA probes marking chicken LBC4, 27 probes demonstrated bright and specific hybridization signal on a corresponding pair of homologous chromosomes in metaphase plates (**Figures 1A–C**). The remainder four probes gave a major hybridization signal on GGA4 as well as additional minor signals dispersed across the karyotype, which might be due to excess of interspersed DNA-repeats in microdissected material. All 5 FISH-probes marking the microchromosome also proved to be bright and very specific (**Figures 1D–F**).

### High-Resolution Mapping and Analysis of Transcriptional Activity of DNA Fragments From Microdissected Chromomeres on Lampbrush Chromosomes

**Figure 2:**
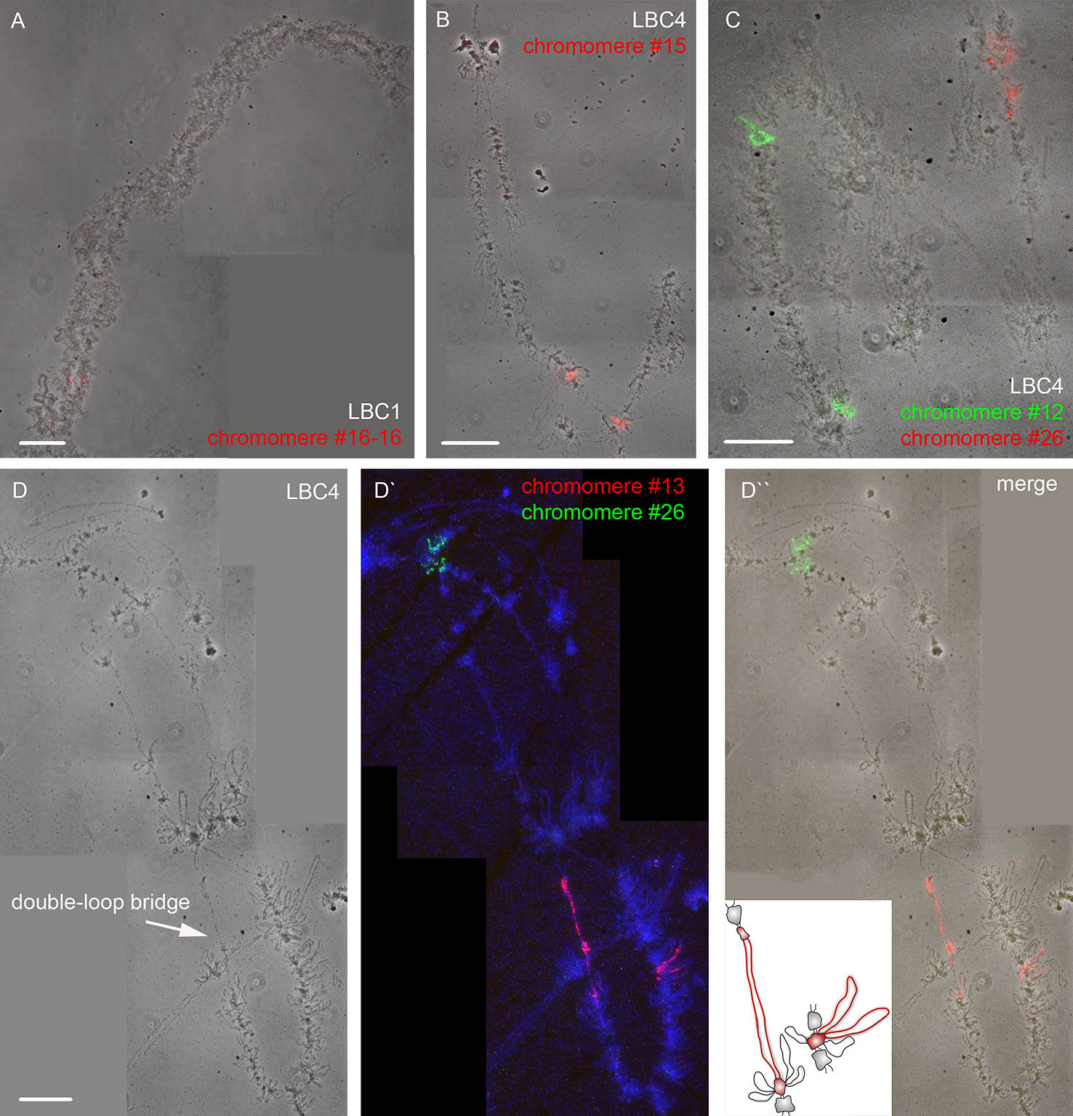
High-resolution FISH-mapping of DNA fragments from microdissected chromomeres on chicken lampbrush chromosomes. Examples of FISH with chromomere DNA probes to LBC 1 **(A)** and LBC 4 **(B, C, D–D'')**. Arrow points to a “double-loop bridge” (DLB) **(D)**; the insert shows a schematic drawing of the DLB region with the mapped FISH-probe to chromomere #13 (red, **D'**). FISH signals are shown on the top of LBC phase contrast images **(A, B, C, D'')**. FISH was carried out according to a DNA/DNA+RNA hybridization protocol. Chromomere ID numbers are indicated. Chromosomes are counterstained with DAPI. Scale bars = 10 μm.

Using a DNA/DNA+RNA hybridization protocol we mapped all DNA sequences from microdissected individual chromomeres on chicken LBCs (**Figure 2**). In most cases, a hybridization signal was observed in a single chromomere similar in size and morphology to the dissected chromomere indicating a tendency of chromomeres to maintain their integrity as individual chromatin domains. At the same time, in some cases we observed a FISH signal in several neighboring chromomeres, which can be explained by different degrees of LBC's condensation during the oocyte growth. That is, chromatin of an individual chromomere dissected from a more compact lampbrush chromosome may be included into several smaller chromomeres in less compact chromosomes.

The majority of DNA probes hybridized to small and medium-sized loose chromomeres, with the hybridization signal being also revealed in RNP-matrix of extended lateral loops (**Figures 2B, C**). Thus we conclude that obtained DNA probes in fact correspond to chromomere-loop complexes of LBC4. In contrast, dissected material of massive marker chromomeres of chicken LBCs 1–3 had been previously revealed in loopless DAPI-positive chromomeres (**Figure 2A**) (Zlotina et al., 2016).

### Investigation of Genomic Context of Individual Lampbrush Chromosome Chromomeres

**Figure 3:**
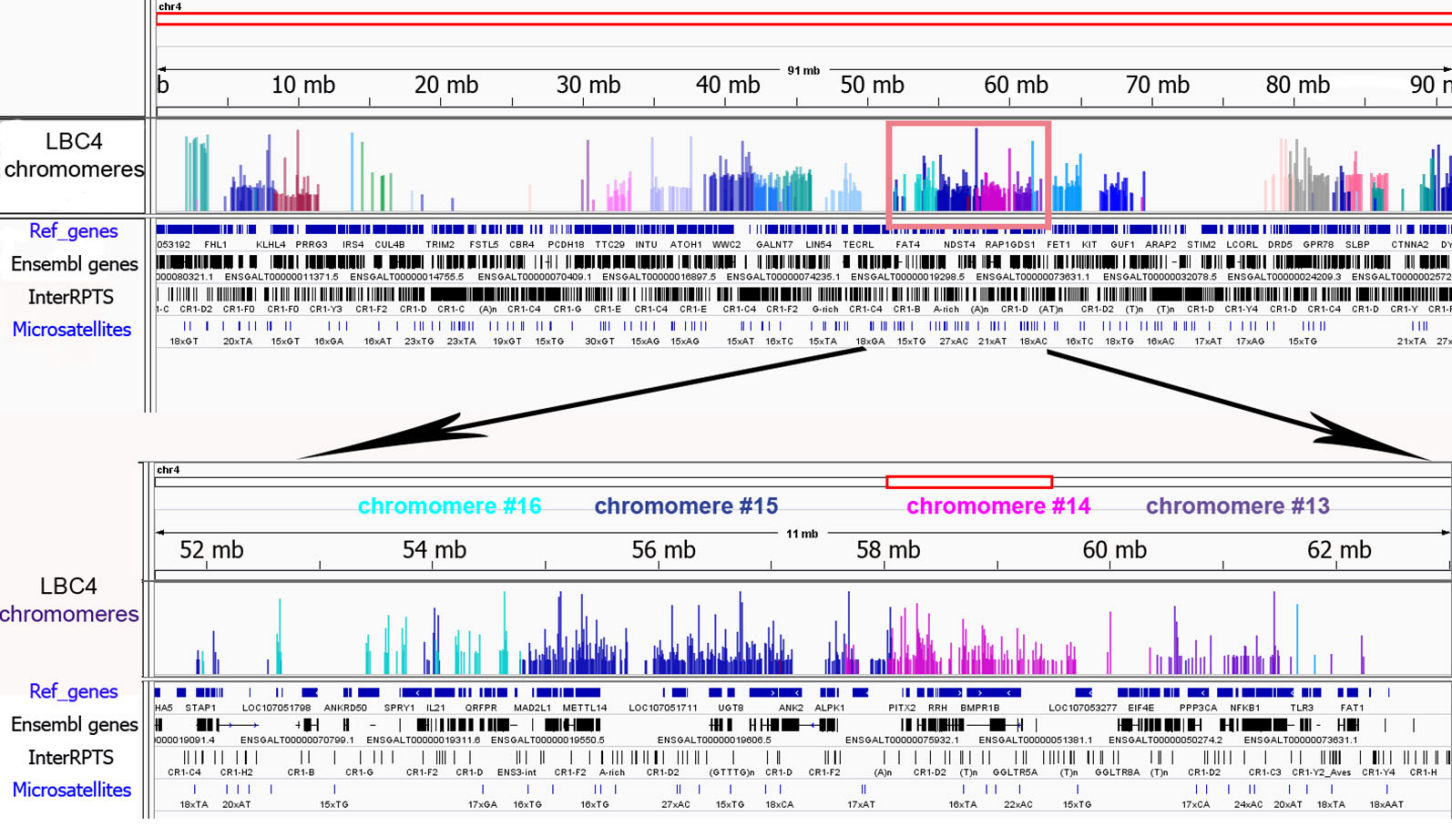
Genomic context of individual lampbrush chromosome chromomeres. Genomic mapping of LBC4 chromomeres to chicken reference genome assembly (Gallus_gallus-5.0). The sequencing data was visualized in the «Integrative Genomics Viewer» (IGV) genomic browser. The sequencing reads corresponding to individual chromomeres are shown in different colors. An upper panel is «the chromosome» view; a lower panel—a zoomed target region encompassing chromomeres #13–16. Imported tracks with annotated genes (Ref_genes, Ensembl genes) and various types of repetitive DNA elements (InterRPTS from the RepeatMasker program which displays interspersed repeats and low complexity DNA sequences, Microsatellites) are shown.

To investigate the genomic context of LBC chromomeres, we applied high-throughput sequencing of individual chromomeres microdissected from chicken lampbrush chromosomes. Earlier we had deciphered several massive DAPI-positive chromomeres dissected from chicken LBCs 1, 2, and 3 (Zlotina et al., 2016). Such marker chromomeres are typical for certain regions of the largest chicken lampbrush marcrochromosomes. In the present study, we sequenced DNA-material of 24 neighboring chromomeres covering the LBC 4 along its length (samples ## macro1–6, 11–23, 27–31), and five chromomeres constituting one of the chicken microchromosomes (samples ## micro1–5). In case of LBC4, all 24 samples of individual chromomere-loop complexes were successfully assigned to GGA4 reference genome assembly with neighboring dissected chromomeres being mapped to adjacent genomic regions (**Figure 3**). Besides, the results of genome mapping allowed identifying the dissected lampbrush microchromosome as chromosome 11.

Further NGS analysis allowed to evaluate the chromomeres’ size and borders. In particular, according to our assessments the amount of DNA in the majority of small and medium-sized chromomeres is about 1.5 to 3.5 Mb, while DNA content of large marker chromomeres is 4 to 5 Mb (**Figure 3**, Zlotina et al., 2016). These results are consistent with previous estimation of chromomere size based on the analysis of cytological maps of a chromomere-loop pattern of LBCs (Galkina et al., 2006).

The microdissected chromomere samples were also analyzed with regard to gene density and repeat content (**Figure 3**). Besides, the sequencing data was compared with the results of FISH-mapping on lampbrush chromosomes. The majority of simple chromomeres had a mixed genomic context and comprised both gene-rich/repeat-poor DNA as well as gene-poor and repeat-rich DNA (for instance, chromomeres of LBC4 ## 11, 14, 15, 19, 20). Based on the FISH data, such DNA sequences were revealed both in chromomere cores and arising lateral loops i.e. chromomere-loop complexes (**Figure 2B**). Some chromomeres demonstrated relatively higher gene density and lower content of repetitive sequences (chromomeres of LBC11, chromomeres #22 and #13 of LBC4). It is worth noting that according to FISH mapping, the DNA probe generated from dissected chromomere #13 hybridized to a so-called «double loop bridge» (a chromosomal region with broken chromomere structure), namely to chromatin fiber and flanking halve-chromomeres (**Figures 2D–D''**). Thus the genomic coordinates of the double loop bridge region were determined precisely, which provides prospects to determine the DNA sequences underlying the formation of such structures. Besides, high-throughput sequencing data demonstrated that DNA of massive loopless chromomeres was significantly enriched by repetitive DNA-elements of different nature and comprised smaller amount of genes as compared to neighboring regions. In particular, the DNA of such chromomeres is enriched by chicken LINE element CR1 (Zlotina et al., 2016).

We conclude that the described complex approach combining cytological, cytogenetic, and genomic analysis allows to correlate morphologically distinct chromatin domains—lampbrush chromosome chromomeres in complex with arising lateral loops—with particular deciphered genomic regions.

### Comparative Analysis of Lampbrush Chromomeres and Chromatin Domains of Interphase Nucleus

Information on genomic coordinates of an array of LBC chromomeres allowed to compare chromomeres with spatial hierarchical chromatin domains earlier characterized by Hi-C in chicken somatic cells (Fishman et al., 2019). In particular, similar to other vertebrates chicken genome proved to be folded into large-scale epigenetically distinct domains: A-compartments containing open and transcriptionally active chromatin and B-compartments with silent chromatin. Within compartments the chromatin is packaged into submegabase-sized topologically associated domains (TADs), which represent local contact-enriched self-interacting chromatin domains.

**Figure 4:**
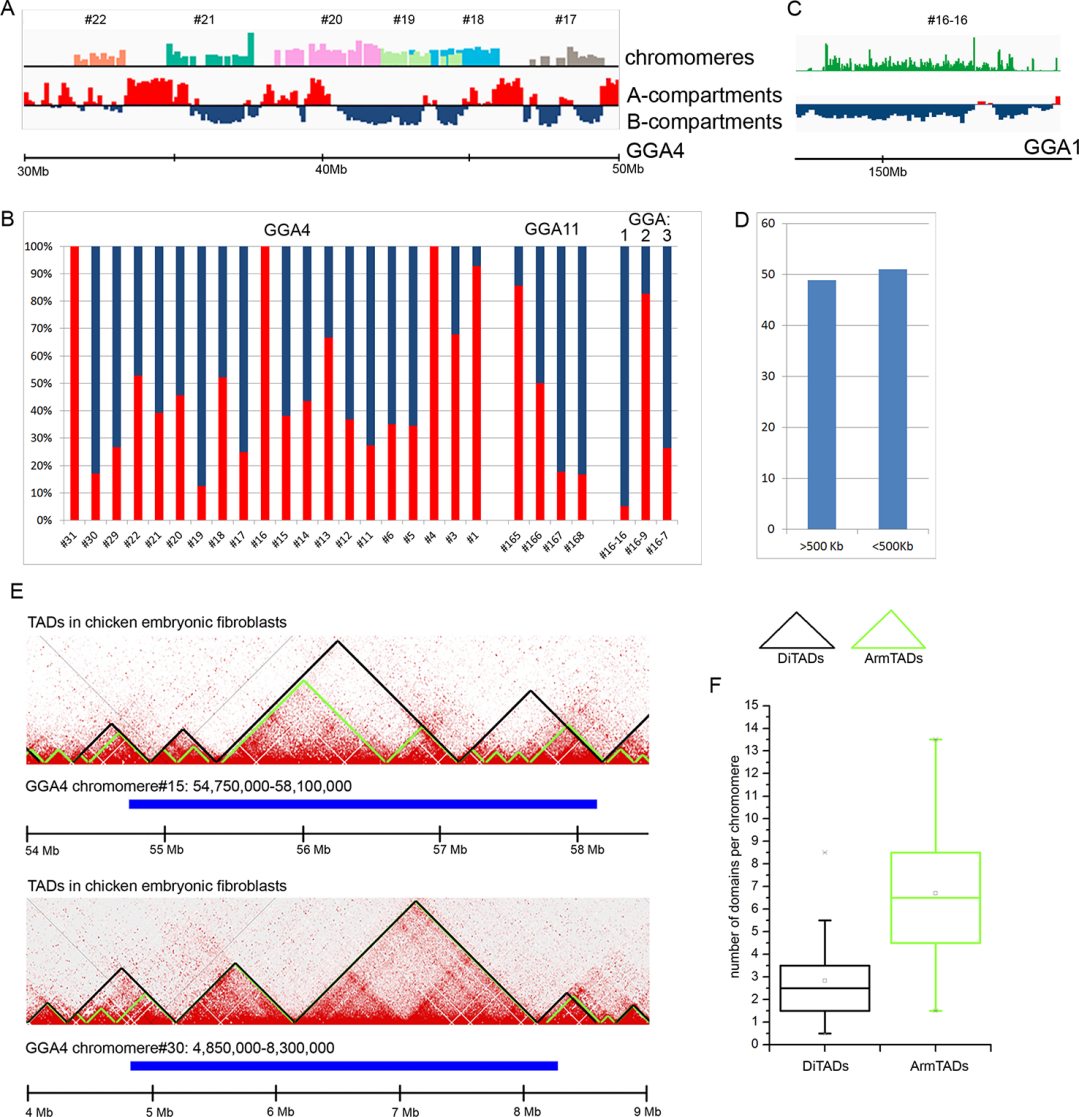
Comparison of chicken lampbrush chromosome chromomeres with A/B compartments and topologically associated domains (TADs) of interphase nucleus. **(A, C)** Alignment of the LBC chromomeres sequences with a profile of A/B-compartments of embryonic fibroblasts. Individual chromomeres are shown in different colors and numbered according to chromomere ID. A- and B-compartments are shown in red and blue, correspondingly. **(B)** The ratio of somatic A- and B-compartments in individual chromomeres from LBC4, LBC11, and LBCs 1–3 (the sequencing data on LBCs 1–3 was described in detail in Zlotina et al., 2016). **(C)** Marker chromomere (#16–16 from LBC1) with pronounced B-status. **(D)** Proportion of switches between the domain type (А→В or В→А) 500 kb upstream or downstream from the right and left chromomere borders. **(E)** Comparison of genomic coordinates of individual LBC chromomeres and somatic TADs. The heatmaps of spatial interactions show TADs, identified by different algorithms: DiTADs (black) and ArmTADs (green). The genomic regions corresponding to LBC chromomeres are shown in blue. **(F)** Boxplots illustrating the number of somatic DiTADs (black) and ArmTADs (green) per chromomere (n = 27).

At first, we compared the genomic regions corresponding to the dissected LBC chromomeres with an A/B compartments profile obtained for chicken embryonic fibroblasts at 100 kb resolution of a contact matrix. By visual matching the genomic coordinates of chromomeres and A/B compartments, we concluded that chromomere borders do not correspond to the boundaries of A/B compartments (**Figure 4A**). In other words, a single chromomere may contain chromatin belonging to both A and B compartments of interphase nucleus. For more thorough analysis, we estimated the ratio of “somatic” A/B compartments in every sequenced chromomere of LBC4 and LBC11 as well as in earlier deciphered marker chromomeres of LBC1, 2, and 3 (**Figure 4B**). The majority of dissected chromomeres contained different proportions of A and B compartments with only single chromomeres being fully overlapped by a compartment of one type. In particular, a prominent marker chromomere from LBC1, which was shown to be gene-poor and highly enriched with repetitive DNA elements (sample #16–16, Zlotina et al., 2016), had an unambiguous «B» status (**Figure 4C**).

Then we analyzed how LBC chromomere borders correlate to the borders of “somatic” A/B compartments (**Figure 4D**). It should be taken into account that genomic borders of microdissected chromomeres were mapped with some precision, which was determined by the accuracy of chromomere identification and isolation during microdissection and by the sequencing depth. We counted switches of the domain type (А→В or В→А) throughout the genomic regions at 500 kb distance from the chromomere borders. We have not found the preferred “switching” of the compartments between A/B types near the chromomeres boundaries. Borders of A/B compartments were found near the chromomere borders in 51% of left and right chromomere borders.

Finally, we compared LBC chromomeres with TADs of chicken somatic cells. We used the genomic coordinates of TADs identified by the directionality index algorithm (DI, Dixon et al., 2012) and Armatus algorithm (Armatus, Filippova et al., 2014) (Fishman et al., 2019). We found that LBC chromomeres generally correspond to several somatic TADs (**Figure 4E**). That is, we analyzed 27 deciphered chromomeres and estimated that one chromomere may comprise from 0.5 to 8.5 DI TADs and from 1.5 to 13.5 Armatus TADs (**Figure 4F**). On the average, chromomeres contained 2.5 DI TADs and 6.5 Armatus TADs.

In general, our data suggest that lampbrush chromosome chromomeres do not correspond unambiguously to any type of spatial genomic domains previously identified in the interphase nucleus of somatic cells.

## Discussion

To get a deeper insight into organization of LBC chromatin domains, we applied the approach that combines mechanical microdissection of individual chromomeres from chicken lampbrush chromosomes, preparation of DNA-libraries from the dissected material followed by high-resolution FISH-mapping and high-throughput sequencing of chromomere DNA (Zlotina et al., 2016). The described approach allowed us to correlate particular chromomere-loop complexes with the deciphered genomic regions.

Until this study, the DNA composition of lampbrush chromosome chromomeres has remained unknown with few exceptions. In particular, some data were obtained for a small number of chromomeres consisting of massive arrays of tandemly repeated sequences. For instance, it was found that the majority of chromomeres on the chicken LBC W are occupied by specific families of DNA repeats (Solovei et al., 1998; Komissarov et al., 2018). Another example includes prominent dense chromomeres in the centromere regions of lampbrush chromosomes that were demonstrated to contain (peri) centromeric DNA repeats (Solovei et al., 1996; Saifitdinova et al., 2001; Krasikova et al., 2006; Krasikova et al., 2012; Zlotina et al., 2019). Additionally, non-centromere clusters of tandem repeats were shown to constitute some interstitial chromomeres (Krasikova et al., 2006; Zlotina et al., 2010). However, as early as in 1980 H. Macgregor suggested that while some chromomeres bear highly uniform DNA (such as clusters of repetitive sequences), the others have a less uniform content (Macgregor, 1980). In the present study, we for the first time established genomic properties of an array of regular chromomeres from chicken LBCs including all morphologically distinct chromomeres from macrochromosome 4 and microchromosome 11. Previously we had also microdissected several individual marker chromomeres from chicken LBCs 1, 2, and 3 (Zlotina et al., 2016). Analysis of the genetic context of all dissected chromomeres allowed us to confirm the Macgregor`s hypothesis. Indeed, we found that regular chromomere-loop complexes generally have a mixed composition and combine genomic regions enriched in genes/depleted for DNA repeats with regions lacking genes/enriched in repetitive elements. At the same time, individual marker DAPI-positive chromomeres typical for the largest chicken LBCs seem to be more homogeneous and demonstrate a high content of repetitive DNA.

Apparently, chromomeres of meiotic lampbrush chromosomes have little in common with chromomeres of polytene chromosomes. In polytene chromosomes that form in interphase nuclei, homologous chromomeres fuse forming a transverse band (Zykova et al., 2018). In Drosophila, the positions of bands and interbands in polytene chromosomes can be predicted by Hi-C technique and confirmed by FISH. Bands and interbands were demonstrated to be equivalent to TADs and the regions between them respectively, with a high degree of conservation between polytene TADs and diploid TADs (Eagen et al., 2015; Ulianov et al., 2016). On the contrary, the data obtained in our study imply that chromomeres of chicken LBCs generally do not correspond to TADs identified in chicken embryonic fibroblasts. In particular, LBC chromomeres are larger structural units of chromatin organization, and genomic regions corresponding to several somatic TADs are involved in their formation. Moreover, along the whole length of GGA4 and GGA11, chromomere borders do not match to the borders of A/B chromatin compartments typical for interphase nuclei of chicken fibroblasts. There are three possible explanations for the lack of correspondence between the boundaries of lampbrush chromomeres and interphase A/B compartments: difference in genomic borders of A/B compartments in interphase nucleus and diplotene oocyte nucleus, the uncertainty of the identification of chromomere borders, or absence of correspondence between the chromomere positions and A/B compartments.

A pattern of transcription during the lampbrush stage of meiosis dramatically differs from one in somatic cells due to a supposed role of LBCs in accumulation of maternal RNAs in growing oocytes. Such a peculiar pattern of transcription leads to a distinctive pattern of untranscribed regions gathering in chromomeres. This can underlay the discrepancy in organization between LBC chromomeres and compact chromatin domains of somatic cells. It was previously suggested that lampbrush chromosome chromomeres appear as a result of massive transcription taking place on the lateral loops (Callan, 1986). That is, lateral loops with RNP-matrix consisting of nascent transcripts and associated RNA-binding factors push apart dense transcriptionally inactive chromatin domains leading to their insularity. The question on the role of CTCF insulator protein in establishing the borders between neighboring LBC chromomeres remains open-ended.

Further single-cell Hi-C studies of oocyte nucleus with a lampbrush chromosome set together with high-resolution FISH-mapping are required to determine chromatin domains with higher frequency of self-interactions and their correspondence to lampbrush chromomeres.

## Data Availability Statement

We have uploaded all DNA sequencing data (for 29 samples) from our study to European Nucleotide Archive (ENA) repository, the study accession number is: PRJEB36087.

## Ethics Statement

The animal study was reviewed and approved by the Ethical committee of Saint-Petersburg State University (approval #131–03-2).

## Author Contributions

AK conceived the study and supervised the project. AK, AZ, NK, AA-R, and TL performed microdissection procedure. AZ planned and carried out most of the principal cytogenetic experiments. AZ and OP performed high-throughput sequencing. AZ and AM performed bioinformatic analysis. AZ, AM, and AK drafted the manuscript. All authors read and approved the final manuscript. 

## Conflict of Interest

The authors declare that the research was conducted in the absence of any commercial or financial relationships that could be construed as a potential conflict of interest.

## Supplementary Material

Supplementary Figure S1 Microdissection of individual chromomeres along the chicken lampbrush chromosome 4. **(A)** Phase-contrast image of chicken LBC 4 subjected to microdissection procedure. Totally, as much as 31 chromomeres were isolated from one of the halve-bivalents starting from the *q*-arm terminal region (chromomeres ##1–31). **(B)** Cytological map of a chromomere-loop pattern of chicken LBC4 (according to [Galkina et al., 2006] with modifications). Cen, centromere position. Arrows point to microdissected chromosome regions.
