# Supplementary figures and images for: New Insights Into Chromomere Organization Provided by Lampbrush Chromosome Microdissection and High-Throughput Sequencing
Source: Front Genet. 2020 Feb 17;11:57. doi: 10.3389/fgene.2020.00057 (PMC7038795; doi:10.3389/fgene.2020.00057)

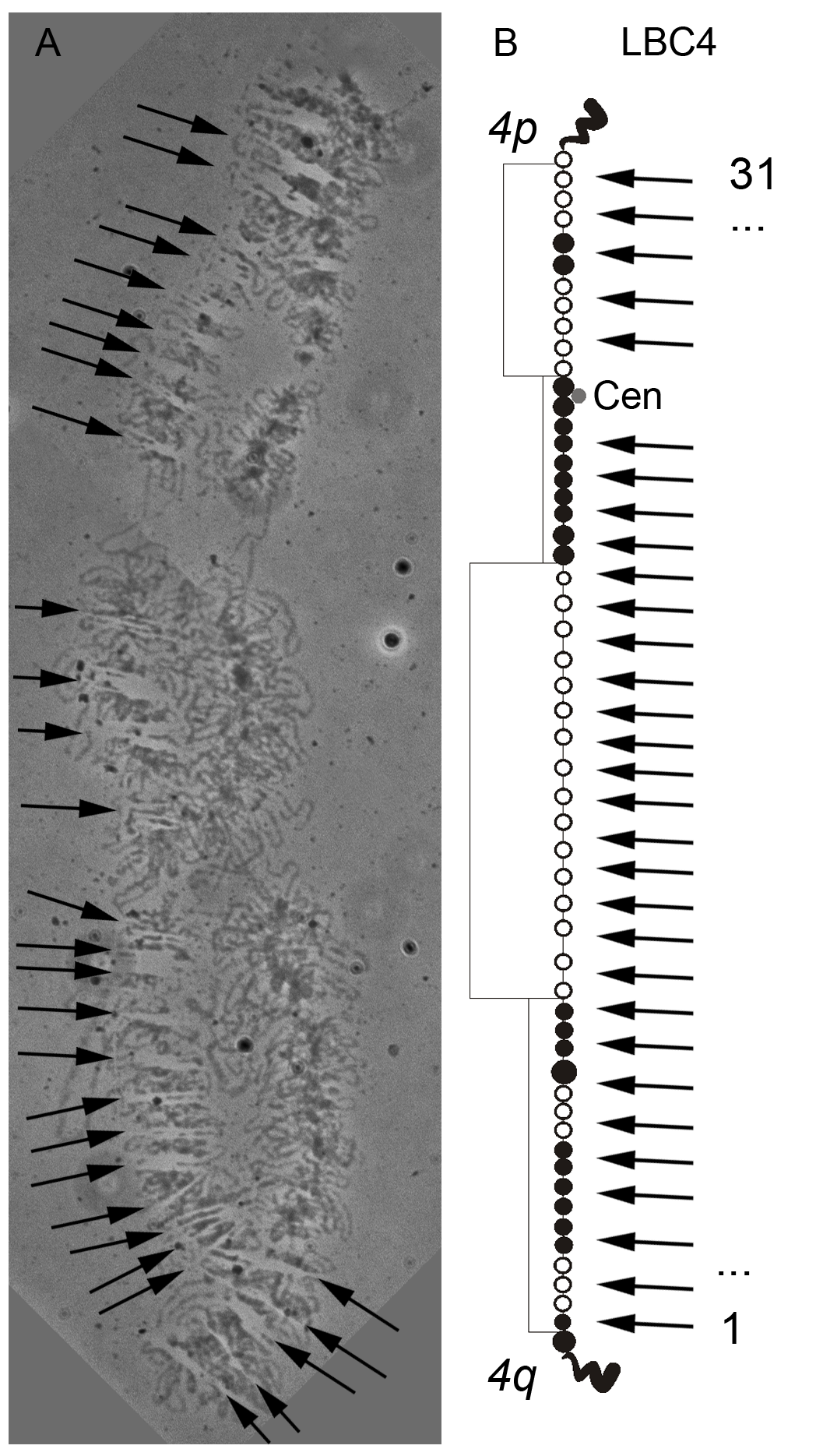

Supplement: Supplementary Figure S1 — Microdissection of individual chromomeres along the chicken lampbrush chromosome 4. (A) Phase-contrast image of chicken LBC 4 subjected to microdissection procedure. Totally, as much as 31 chromomeres were isolated from one of the halve-bivalents starting from the q-arm terminal region (chromomeres ##1–31). (B) Cytological map of a chromomere-loop pattern of chicken LBC4 (according to [Galkina et al., 2006] with modifications). Cen, centromere position. Arrows point to microdissected chromosome regions. [file Image_1.tif]
